# Connectional subdivision of the claustrum: two visuotopic subdivisions in the macaque

**DOI:** 10.3389/fnsys.2014.00063

**Published:** 2014-05-07

**Authors:** Ricardo Gattass, Juliana G. M. Soares, Robert Desimone, Leslie G. Ungerleider

**Affiliations:** ^1^Program of Neurobiology, Institute of Biophysics Carlos Chagas Filho, Universidade Federal do Rio de JaneiroRio de Janeiro, Brazil; ^2^Laboratory of Neuropsychology, National Institute of Mental Health, National Institutes of HealthBethesda, MD, USA; ^3^McGovern Institute for Brain Research at MITCambridge, MA, USA; ^4^Laboratory of Brain and Cognition, National Institute of Mental Health, National Institutes of HealthBethesda, MD, USA

**Keywords:** V4, visual topography, cortical connections, integration of visual maps, cross-modal association

## Abstract

The claustrum is a surprisingly large, sheet-like neuronal structure hidden beneath the inner surface of the neocortex. We found that the portions of the claustrum connected with V4 appear to overlap considerably with those portions connected with other cortical visual areas, including V1, V2, MT, MST and FST, TEO and TE. We found extensive reciprocal connections between V4 and the ventral portion of the claustrum (vCl), which extended through at least half of the rostrocaudal extent of the structure. Additionally, in approximately 75% of the cases, we found reciprocal connections between V4 and a more restricted region located farther dorsal, near the middle of the structure (mCl). Both vCl and mCl appear to have at least a crude topographic organization. Based on the projection of these claustrum subdivisions to the amygdala, we propose that vCl and mCl are gateways for the transmission of visual information to the memory system. In addition to these crude visuotopically organized regions, there are other parts of the claustrum that obey the topographical proximity principle, with considerable overlap of their connections. There is only an overall segregation of claustrum regions reciprocally connected to the occipital, parietal, temporal and frontal lobes. The portion of the claustrum connected to the visual cortex is located ventral and posterior; the one connected to the auditory cortex is located dorsal and posterior; the one connected to the somatosensory cortex is located dorsal and medial; the one connected to the frontal premotor and motor cortices is located dorsal and anterior; while the one connected to the temporal cortex is located ventral and anterior. The extensive reciprocal connections of the claustrum with almost the entire neocortex and its projections to the hippocampus, amygdala and basal ganglia prompt us to propose its role as a gateway for perceptual information to the memory system.

## Introduction

The claustrum is a thin, irregular, sheet-like neuronal structure hidden beneath the inner surface of the neocortex. It is a very narrow nucleus (1–6 mm), except for its ventral portion (3–16 mm). Laterally, it wraps around the basal nuclei, mainly putamen (and a very small portion of the anterior thalamus). It resembles a leaf with two segments, one extending anteriorly into the frontal lobe and the other extending anteriorly into the temporal lobe. A lateral reconstruction of the claustrum reveals that this nucleus is surprisingly large in its anterior-to-posterior extent (30 × 20 mm). There was a debate concerning the ontogenetic origin of the claustrum, with three different opinions being argued: that the structure is derived from the adjacent insular cortex (Meynert, 1868), that it is part of the basal ganglia (Edelstein and Denaro, 2004), and that the claustrum does not have cortical or subcortical origins (Filimonoff, 1966). A more recent proteomics study of the rat claustrum agreed with this third view. The authors found that the claustrum is an intermediate structure between the striatum and the cortex, although having an affinity with layer VI of the insular cortex (Mathur et al., 2009). We favor its pallidal origin based on the mapping of transcription factors and data from its early morphogenesis (Edelstein and Denaro, 2004).

Crick and Koch (2005) summarized what was known about the claustrum, and speculated on its possible relationship with the processes that give rise to integrated conscious percepts. The portions of the claustrum connected with V4 appear to overlap considerably with portions that are connected to other visual cortical areas, including V1 (Mizuno et al., 1981; Doty, 1983), V2 (Pearson et al., 1982), MT (Maunsell and van Essen, 1983; Ungerleider et al., 1984), MST and FST (Boussaoud et al., 1992), TEO (Webster et al., 1993), and TE (Nauta and Whitlock, 1956; Kemp and Powell, 1970; Turner et al., 1980; Baizer et al., 1993; Webster et al., 1993). Evidence in other species suggests that the claustrum may be specialized for visuomotor tasks due to its connections with the different visual and motor subdivisions of the cortex (Olson and Graybiel, 1980). Based mainly on findings from a study using 2-Deoxy-d-glucose (2-DG), Ettlinger and Wilson (1990) speculated that the claustrum is involved in cross-modal associations. We found extensive reciprocal connections between V4 and vCl that extended through at least half of the rostrocaudal extent of the structure (Gattass et al., 2014). Additionally, in approximately 75% of the cases, we found reciprocal connections between V4 and a more restricted region in the claustrum located farther dorsal, near the middle of the structure (mCl). Both vCl and mCl appear to have a crude topographic organization, based on the visuotopic location of the V4 injection sites. Based on the projection of these portions of the claustrum to the amygdala (Turner et al., 1980), we propose that vCl and mCl are gateways for the transmission of visual information to the memory system.

In this study, we review the total extent of the connections of V4 with the claustrum, as well as their topographic organization in the context of the connections of the claustrum with other cortical areas. We describe the overall extent of the claustrum in one macaque and its connections in nine macaque monkeys with combined tritiated amino acid (^3^H), wheat germ agglutinin conjugated to horseradish peroxidase (HRP) and retrograde fluorescent tracer injections, placed under physiological control, into 19 different retinotopic locations of V4. We compare the organization of these visual regions of the claustrum with other portions of the claustrum connected with other sensory, motor, and association cortices.

## Materials and methods

**Figure 1:**
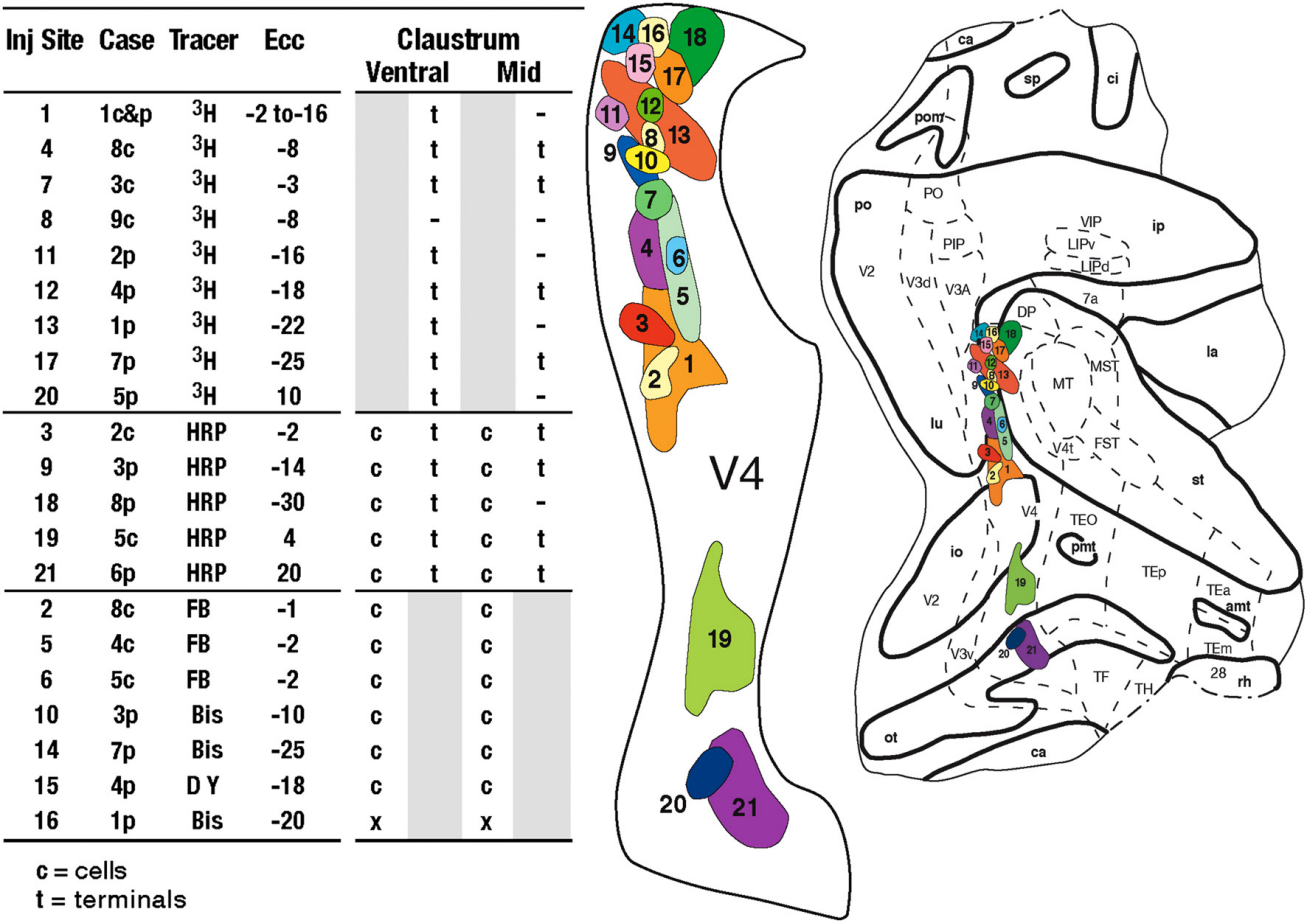
***Left:*** Table with the summary of the projections from and to V4. c, cells; t, terminals; gray areas, not applicable for the tracer; –, relevant sections with no label; x, relevant sections not analyzed; ECC, eccentricity of the injection. ***Right:*** Injection sites in V4 shown in a flattened map of extra-striate cortex. Tracers were placed in 21 injections sites at central and peripheral locations in V4, in 9 animals (cases) in 10 hemispheres. Each injection site is numbered and colored to match data from other figures. Myeloarchitectonic borders of visual areas are indicated with dashed lines. The injections from the individual cases were plotted on this map to best retain their locations relative to myeloarchitectonic borders and sulci. Abbreviations: *Cortical visual areas:* FST, visual area FST; LIPd, dorsal portion of lateral intraparietal area; LIPv, ventral portion of lateral intraparietal area; MIP, medial intraparietal area; MST, medial superior temporal area; MT, visual area MT; MTp, peripheral portion of MT; PIP, posterior intraparietal area; PO, parieto-occipital area; TEm, medial portion of area TE; TEO, posterior inferior temporal cortex; TEp, posterior portion of area TE; TH, cytoarchitectonic area TH; V1, primary visual cortex; V2, second visual area; V3A, visual complex V3, part A; V3d, dorsal portion of visual area 3; V3v, ventral portion of visual area 3; V4, visual area 4; V4t, V4 transition zone; VIP, ventral intraparietal area; VTF, visual portion of parahippocampal TF; *Claustrum:* fCl, frontal subdivision of the claustrum; tCl, temporal subdivision of the claustrum; pCl, parietal subdivision of the claustrum; mCl, mid claustrum; vCl, ventral claustrum; *Cortical sulci:* amt, anterior middle temporal sulcus; ar, arcuate sulcus; ca, calcarine fissure; ce, central sulcus; ec, external calcarine sulcus; io, inferior occipital sulcus; ip, intraparietal sulcus; la, lateral sulcus; lu, lunate sulcus; ot, occipitotemporal sulcus; p, principal sulcus; po, parietal occiptal cleft; pmt, posterior middle temporal sulcus; rh, rhinal sulcus; sp, subparietal sulcus; st, superior temporal sulcus.

The shape and location of the claustrum in the macaque was determined in a series of Nissl-stained coronal sections from an adult *Macaca mulatta*. ^3^H, HRP, and the fluorescent tracers fast blue (FB), diamidino yellow (DY), and bisbenzimide (Bis) were injected in 10 hemispheres of 9 adult *Macaca mulatta*, weighing between 3.2 and 4.4 kg. In all animals, the injections of the tracers were placed into retinotopically specified sites (*n* = 21) in V4, which were determined by electrophysiological recordings. The injection sites, two or more in each animal, spanned eccentricities from central to peripheral vision (Figure 1) in both the upper (*n* = 3) and lower (*n* = 18) visual fields (Gattass et al., 1988).

### Receptive field recording

All experimental procedures were approved by the NIMH Animal Care and Use Committee and described in detail previously (Gattass et al., 2014). The procedures for multi-unit recordings and cortical injections have been reported in detail elsewhere (Gattass and Gross, 1981). Briefly, prior to the first recording session, under ketamine and sodium pentobarbital anesthesia, the animal was implanted with a bolt that was used to hold its head in the stereotaxic apparatus and the stainless steel recording chamber. During each recording session, the animal was anesthetized with 2% halothane, followed by a mixture containing 70% N_2_O and 30% O_2_. Muscular paralysis was induced by pancuronium bromide, and a respiratory pump connected to an endotracheal tube maintained artificial ventilation. The level of CO_2_, heart rate, and rectal temperature were continuously monitored and kept within the normal physiological range. The right eye was protected by a contact lens that focused the eye on the surface of a 57-cm radius translucent hemisphere placed in front of the animal. The locations of the fovea and the center of the optic disc were projected onto the hemisphere using the target of an ophthalmoscope reflected by a corner cube prism (Edmund Scientifics, Barrington, New Jersey). The horizontal meridian was considered a line through both these points, and the vertical meridian was an orthogonal line that passes through the fovea.

Prior to the injections, we mapped the pertinent portion of V4 with the aid of varnish-coated tungsten microelectrodes. The electrodes were assembled in a micromanipulator that could be used to record from small clusters of neurons or could hold a pre-aligned micro syringe to deliver the anatomical tracer. Visual receptive fields were plotted by moving manually white or colored 2D-bars onto the surface of the translucent hemisphere, under light-adapted conditions. Recordings continued until the desired visual field representation within V4 was located.

### Injections of V4

We injected anterograde and retrograde tracers into 21 sites in 9 macaques under electrophysiological guidance. Pressure injections into the cortex were done using a 1-μl Hamilton syringe with a beveled 27-gauge needle that was guided into the appropriate site with the aid of an operating microscope. Sulcal and gyral landmarks were used to identify the location of area V4 (Zeki, 1978; Gattass et al., 1988). In six animals, injections were placed at physiologically determined sites on the prelunate gyrus under direct visualization of the cortex. In the remaining three animals, after the desired injection site was located electrophysiologically, a guide tube was inserted through the dura and placed approximately 300 μm above the intended injection site. The microelectrode was then advanced through the guide tube and the visuotopic location of the injection site was confirmed. The electrode was then withdrawn from the guide tube and replaced with a 1-μl Hamilton syringe. For the remainder of the paper, we refer to each injection site as a case.

In 9 cases, we injected 0.15–0.3 μl of a 1:1 mixture of tritiated proline (New England Nuclear L-[2,3,4,5-3H], specific activity 100–140 Ci/mmol), and tritiated leucine (New England Nuclear L-[3,4,5-3H(N)], specific activity 100–140 Ci/mmol). The labeled amino acids, which had been evaporated and then reconstituted in 0.9% saline to give a final concentration of 50 μCi/μl, were injected at the rate of 0.02 μl/2 min. To minimize leakage of the tracer up the electrode track, the syringe was left in place for 30 min after the injection and then withdrawn into the guide tube, which was then removed from the brain. In 7 cases, one to three injections (0.15–0.3 μl each at each site) of aqueous solutions of 2% FB, 4% DY, or 10% Bis were placed in V4. In 5 cases, two to four injections (0.2 μl each) of 5% of HRP were placed in V4. In the animals with injections involving both HRP and other tracers, the other tracer(s) were injected into the designated V4 sites during one procedure; then, 4–6 days later, HRP was injected into another V4 site.

The amount, concentration, and liquid vehicle of the tracer injections as well as the survival times were selected to produce anterograde and retrograde labeling of equivalent size. However, the nature of the tracers caused small differences in sensitivity. Among the tracers used, HRP was the most effective as both an anterograde and a retrograde tracer. Among the fluorescent dyes, the most effective retrograde tracer was FB, which was closely followed by DY.

### Histological processing

After survival times of 2 days for HRP and 6–8 days for the other tracers, the animals received a lethal dose of sodium pentobarbital and were then transcardially perfused with 0.9% saline followed by 10% formaldehyde-saline. The brains were blocked with the aid of a stereotaxic apparatus, removed from the skull, photographed, and stored in 30% sucrose in 10% formaldehyde-saline until they sank. Frozen sections, 33 μm in thickness, were cut in the frontal plane. One case (Case 6) was cut in the parasagittal plane. Every fifth section was mounted onto gelatinized slides, dehydrated, defatted and processed for autoradiography according to the procedures of Cowan et al. (1972). These sections were dipped in Kodak NTB2 emulsion and exposed at 4°C for at least 12 weeks. Subsequently, the autoradiographs were developed in Kodak D19, fixed, and counterstained with thionin. Another series of sections, which were 250 μm apart, was processed for HRP histochemistry according to a modified tetramethylbenzidine protocol (Gibson et al., 1984). Of these sections, one in four (i.e., 1 section for each mm) was counterstained with thionin, whereas the remaining sections were left unstained and were coverslipped. Anterograde and retrograde labeling was plotted on enlarged photographs (10×) of the myelin-stained and/or thionin-stained sections for subsequent analysis. The boundaries of the various thalamic nuclei were determined from the thionin-stained sections. The Atlas of Olszewski (1952) was used as a reference for nomenclature and for delineating the thalamic boundaries. The locations of concentrations of silver grains, HRP-labeled cells and terminals and fluorescent-labeled cells were assigned to specific subcortical structures in each animal and then combined to evaluate the topographical organization of the connections. Alternate sections were stained for myelin according to the Gallyas' (1979) method.

## Results

In this section, we show the size, shape and location of the claustrum in the macaque and we revisit the data on the connections of the claustrum with V4 to reveal the location and organization of two visuotopic organized regions in the claustrum that are connected to virtually all visual areas of the occipital, temporal, and parietal lobes.

### Shape and location of the claustrum in coronal sections

**Figure 2:**
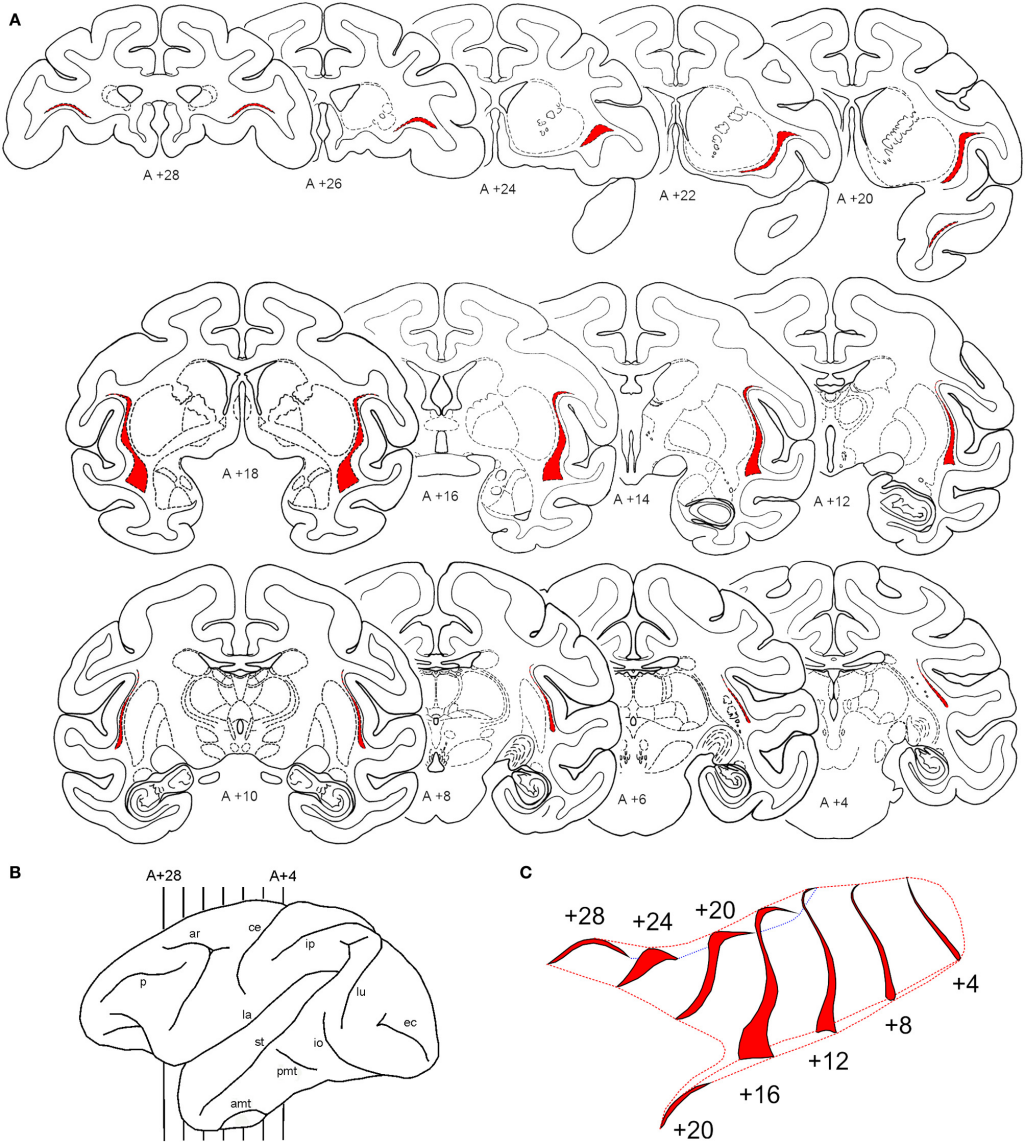
**Shape and location of the claustrum**. Cororonal sections stained for Nissl through the rostral-to-caudal (top-to-bottom) extent of the claustrum. Series of coronal sections, 2 mm apart, with the claustrum shown in red **(A)** from the levels A +28 to A +4 shown in the lateral reconstruction of the left hemisphere **(B)**. An elongated 3D representation in the anterior-to-posterior dimension of the claustrum surface is shown in **(C)**. For abbreviations see legend for Figure 1.

The claustrum is a narrow nucleus (1–6 mm in most of its extent, and 3–16 mm in its ventral portion) that laterally wraps around the basal nuclei, mainly putamen and a very small portion of the anterior thalamus (see Figure 2). For most of its extent the claustrum is lateral to the putamen and medial to the insular cortex. It resembles a leaf with two segments, one extending anteriorly, into the frontal lobe and the other extending anteriorly into the temporal lobe. A lateral reconstruction of the claustrum reveals that this nucleus is surprisingly large in its anterior-to-posterior extent and typically extends over 30 mm. Figure 2 shows the shape and location of the claustrum in coronal sections extending from A + 28 to A + 4 mm in one animal. To better visualize this narrow nucleus, we show the regions containing densely packed cells in red in sections spaced 2 mm apart (Figure 2A). The lateral view of the hemisphere (Figure 2B) shows its anterior-to-posterior extent from the level anterior to the arcuate sulcus to the posterior portion of the central sulcus. To illustrate its complex convoluted surface, we reconstructed the claustrum every 4 mm with additional spaces in the rostrocaudal dimension (Figure 2C). Using an elongated view in this dimension, one can see the convexity of the claustrum and its lateral fold that wraps around the insular cortex (lateral sulcus). Ventrally, the claustrum presents a broader base that extends into the temporal pole (Figure 2, A +20).

**Figure 3:**
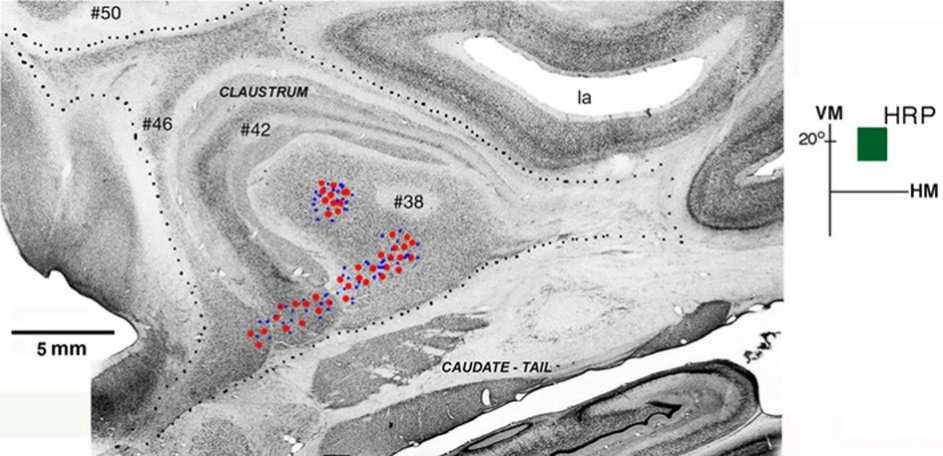
**Connections of V4 with the claustrum in a photomontage of parasagittal sections of Case 6p**. Retrogradely labeled cells (*red circles*) and/or anterogradely labed terminals (*blue dots*) were found in two areas of the claustrum after injection of HRP in the upper field representation of V4 (*dark green square, in insert*). Four parasagittal sections were cut, aligned and stacked to reconstruct most of the extent of the nucleus showing two patches of labeled cells in ventral (*vCl*) and mid (*mCl*) claustrum. Black dots mark the external boundary of the claustrum. VM, vertical meridian; HM, horizontal meridian; la, lateral sulcus. Scale bar: 5 mm.

The shape and location of the claustrum is also shown in a photomontage of parasagittal sections of Case 6p (Figure 3). Four parasagittal sections were cut, aligned and stacked to reconstruct most of the claustrum. The more medial section (#50) shows the extent of the claustrum into the frontal pole. The next section (#46) shows the cells of the claustrum bridging from the frontal to the temporal pole. More lateral sections (#42 and #38) show more central portions of the nuclei. This parasagittal photomontage shows a sparser group of cells in the tail of the caudate, underneath the lateral sulcus. Overall, the anterior-to-posterior extent of the nucleus in this animal is larger than the one illustrated in Figure 2.

**Figure 4:**
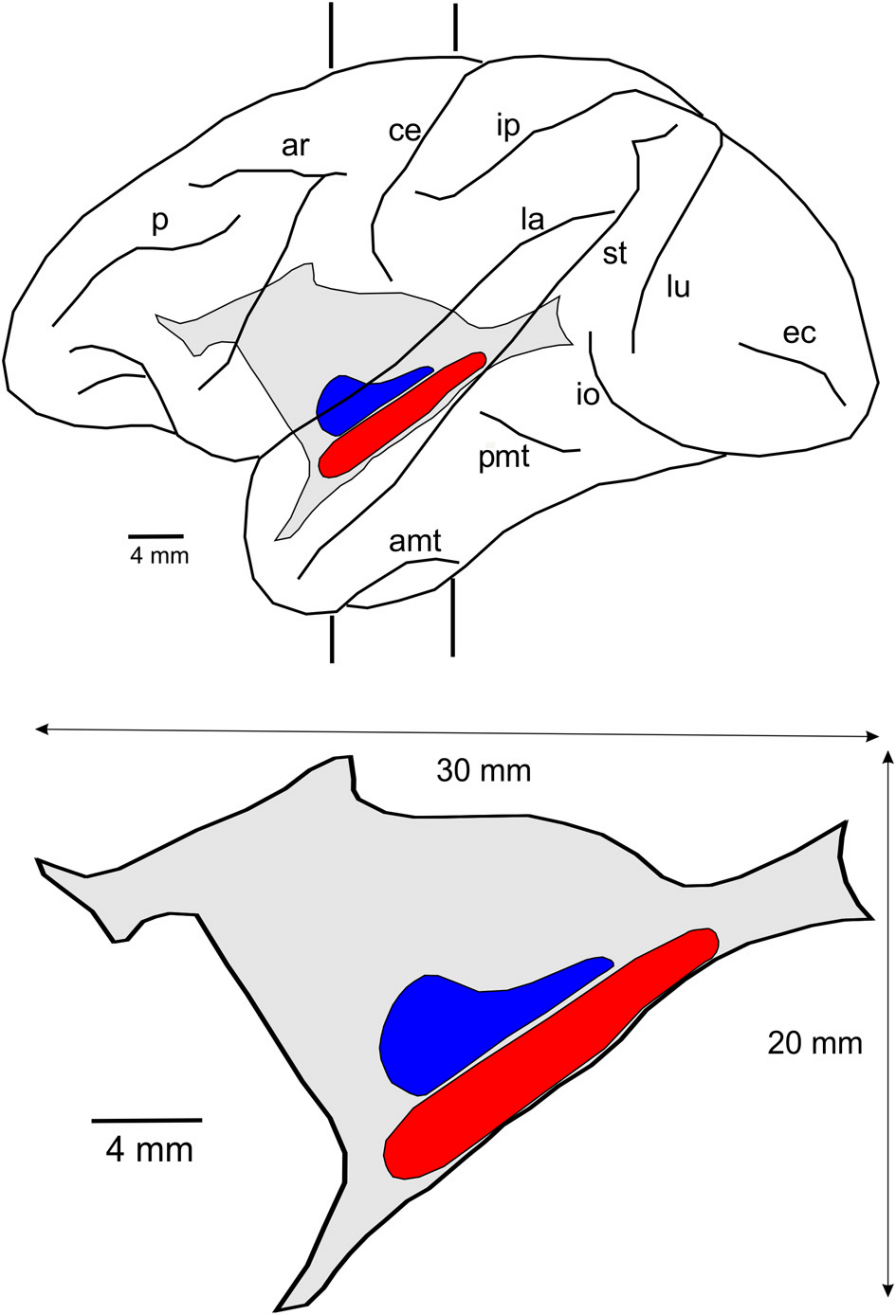
**Location and size of the claustrum**. Lateral reconstruction of the left hemisphere (upper) containing the reconstruction of the claustrum with the two regions connected with V4 and other extrastriate visual areas. The contour of the lateral projection of the claustrum is shown below with an indication of its approximate dimension and the location of the two visuotopically organized regions, vCl (red) and mCl (blue). For abbreviations see legend for Figure 1.

The lateral reconstruction of Case 6p and the lateral projection of the claustrum in this animal are shown in Figure 4. The size and location of this nucleus reveals that the surface of the claustrum is approximately 30 × 20 mm. This figure also shows the location of the two visuotopically organized regions of the claustrum, named vCl (shown in red) and mCl (shown in blue).

### Evidence for two coarse visual topographical areas

**Figure 5:**
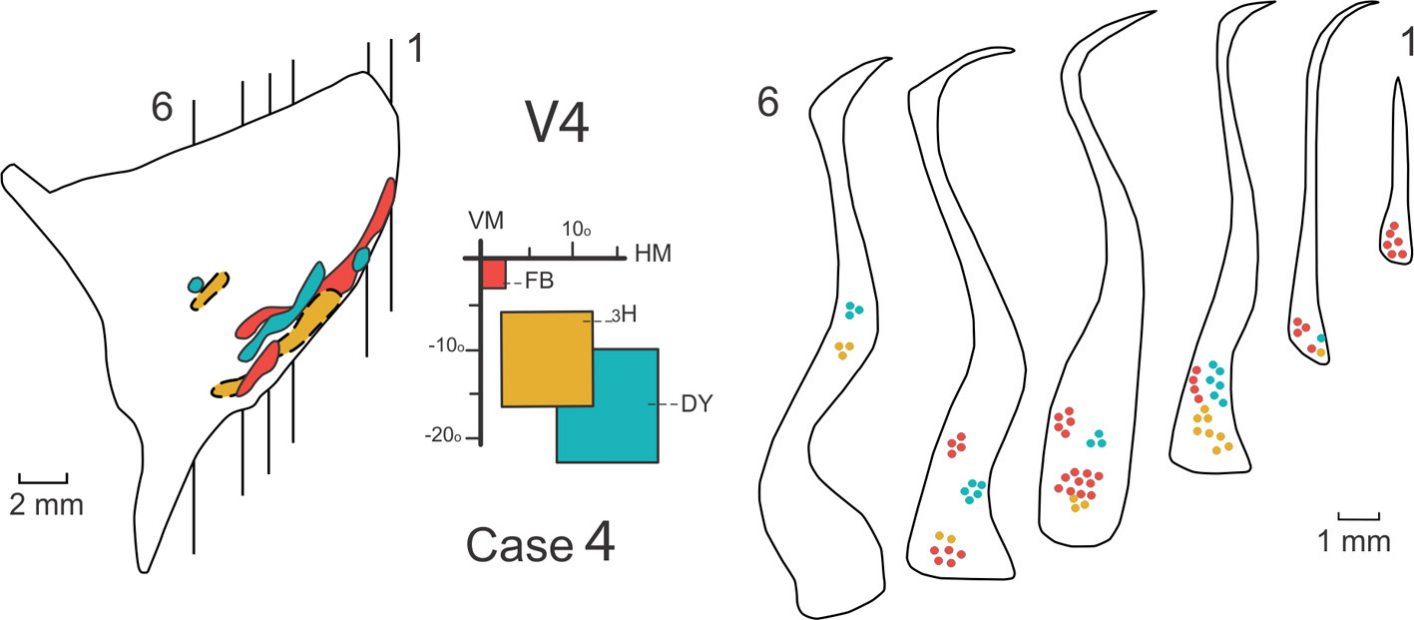
**Connections of the claustrum with area V4**. Afferent and efferent connections of V4 to the claustrum are shown in 6 coronal sections at the levels indicated in the lateral reconstruction of the claustrum in Case 4. The projections from central (red), intermediate (yellow), and peripheral (blue) lower field representations of V4 are segregated into two areas of the claustrum, a medial anterior one and a ventral one. For details see text.

**Figure 6:**
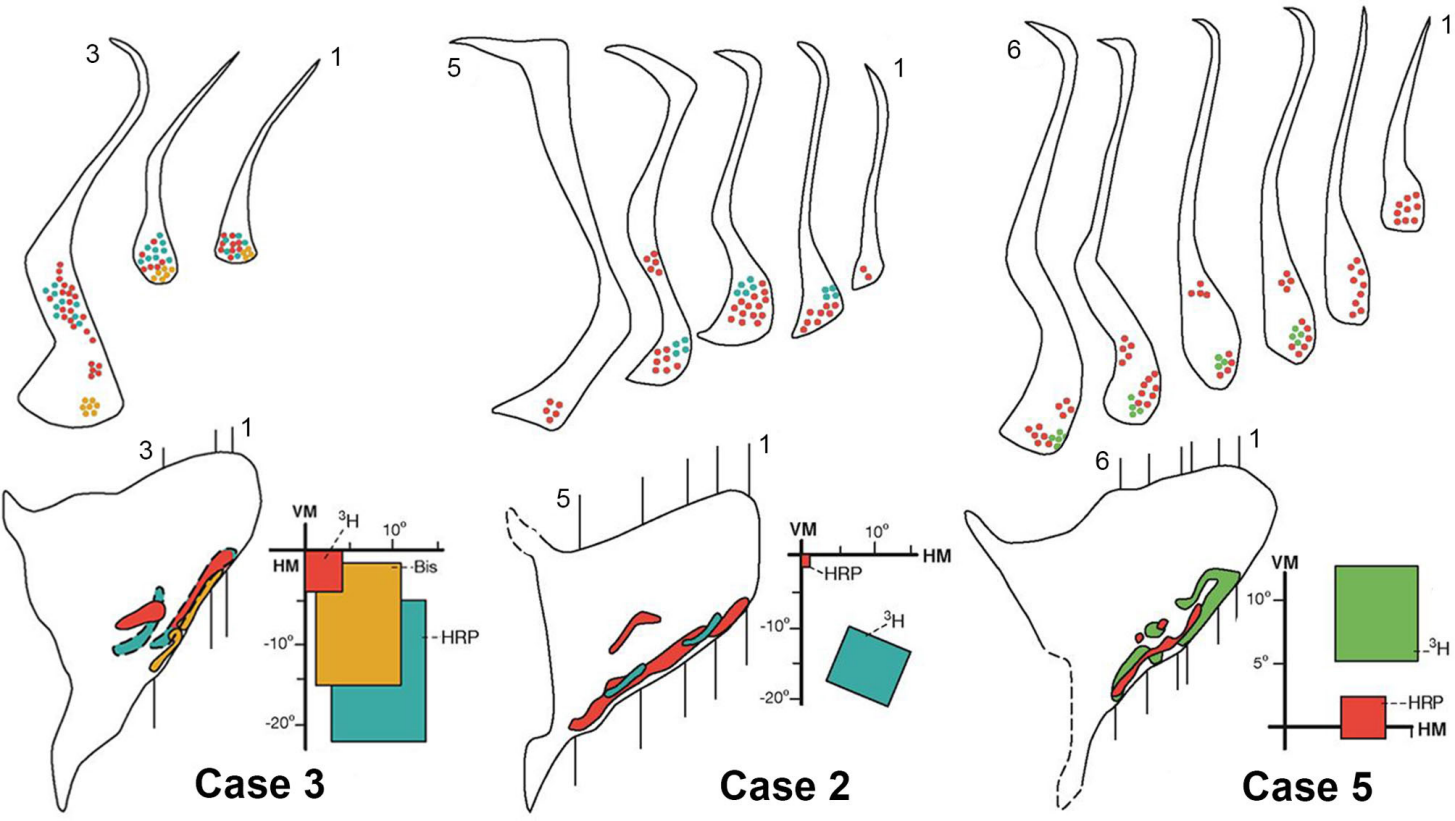
**Connections of the claustrum with area V4**. Afferent and efferent connections of V4 with the claustrum are shown in coronal sections at the levels indicated in the lateral reconstruction of the claustrum in three representative cases: 2, 3, and 5. The projections from central (red), intermediate (yellow), and peripheral lower (blue) or upper (green) field representations of V4 are segregated into two areas of the claustrum, vCl and mCl. For details see text.

From the 21 sites injected in V4, two sites (one in Case 1, site 8 and another in Case 9, site 16) did not reveal projections to the claustrum (see Figure 1). Thus, 19 injections in V4 revealed two areas with crude visual topography one located in ventral portion of the claustrum (vCl) and the other in mid (mCl) claustrum. The data are compatible with a crude and variable topography from animal to animal, but overall there are definite indications of topography, especially in more anterior portions of the claustrum. The visual topography of these areas is best seem in sections viewed in the coronal plane, inasmuch as they overlap in the lateral reconstruction. We choose to show the visual topography by showing cases with 2–3 injections at different eccentricities in V4 (Figures 5, 6). First we show a case with a single upper field injection (Figure 3).

Figure 3 shows the result of an injection of HRP in the upper field representation (+20°) of V4 in Case 6p. Two patches of labeled cells and terminals were observed, one in ventral (vCl) and the other in mid (mCl) claustrum, showing that the upper field representation of V4 is reciprocally connected with these two regions. Retrograde labeled cells (red circles) and anterogradely labeled terminals (blue dots) were found in two segregated clusters after an injection of HRP into V4. The ventral region (vCl) had cells and terminals that appeared in three parasagittal sections (#38, #42, and #46) stacked to reconstruct most of the extent of the nucleus. Cells and terminals in the mid-region (mCl) only appeared in section #38.

Figure 5 shows the result of injections of three different tracers: FB, ^3^H, and DY in the central, intermediate and peripheral representations of the lower visual field in V4 in Case 4. Central (red) and peripheral (blue) injections reveal feedback projections from the claustrum to V4, while the intermediate injection (yellow) reveals feedforward projections to the claustrum. Six representative coronal sections show the feedback and feedforward projections in the claustrum. The lateral reconstruction of the claustrum shows that these projections are contiguous in the ventral portion of the nucleus. The projections are spatially segregated and occupy both the ventral claustrum (vCl) and the mid-claustrum (mCl). An expansion of the central field representation was observed in vCl.

Figure 6 shows the results of the central, intermediate and peripheral injections of the different tracers in the lower field representation of V4 in Cases 2 and 3 and in the upper field representation in Case 5. Injections of ^3^H, Bis, and HRP in central (red), intermediate (yellow) and peripheral (blue) representations of the lower field of V4 in Case 3 revealed feedback and feedforward projections into two regions of the claustrum in three representative coronal sections. The receptive fields corresponding to the injections significantly overlap and the projections are poorly segregated (Figure 6 left). Injections of HRP and ^3^H in central (red) and peripheral (blue) representations of the lower field in V4 revealed segregated patches in the ventral portion of the claustrum and a central patch in the mid-claustrum. These bidirectional connections are shown in 5 representative coronal sections and illustrated on the lateral reconstruction of the claustrum in Case 2 (Figure 6, middle). The injections of HRP and ^3^H in the central and peripheral upper field of V4 in Case 5 are shown in Figure 6 (right). The resulting projections are shown in 6 representative coronal sections, illustrating a clear segregation in the ventral portion of the claustrum. The lateral reconstruction of the claustrum revealed that the upper field in mid-claustrum is represented ventrally, closer to the upper field representation of vCl.

### Coarse visual topography of bidirectional connections

**Figure 7:**
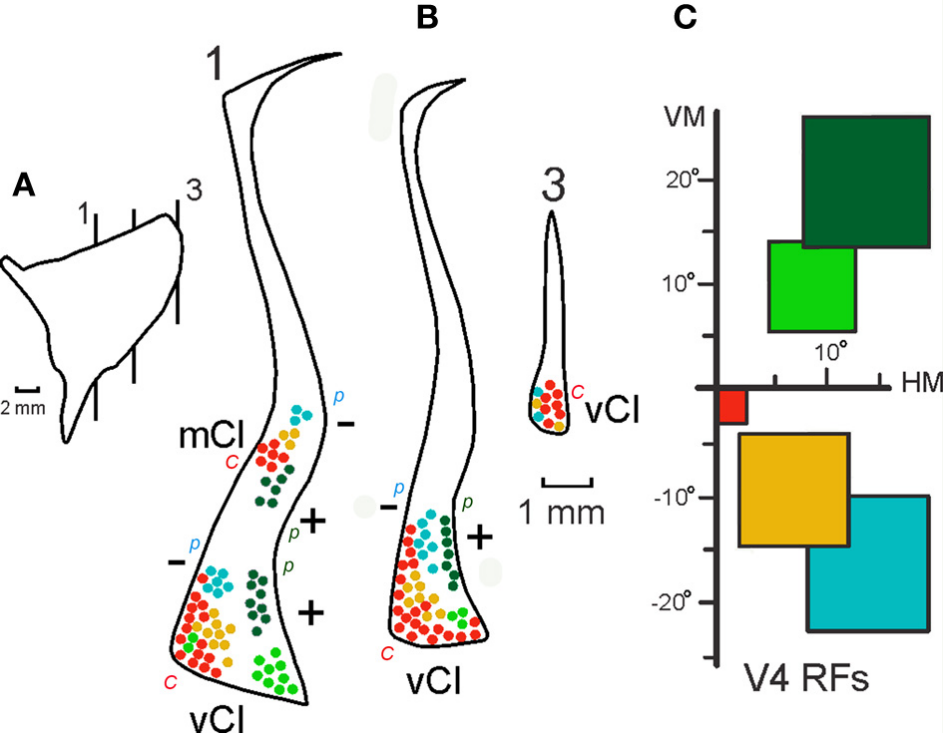
**Intermediate summary data showing the combined projections of 12 injections in V4 onto three representative sections at different levels of the claustrum**. Three sections **(B)** at the level indicated in the lateral reconstruction of the claustrum **(A)** show the projections of injections at central, intermediate and peripheral upper and lower field representations in V4 **(C)**. The first section shows portions of mCl and vCl and details of the visual topography depicted in the next figure. *Labels:* c, central field representation; p, peripheral field representation; +, upper visual field; −, lower visual field representation. For details see text.

**Figure 8:**
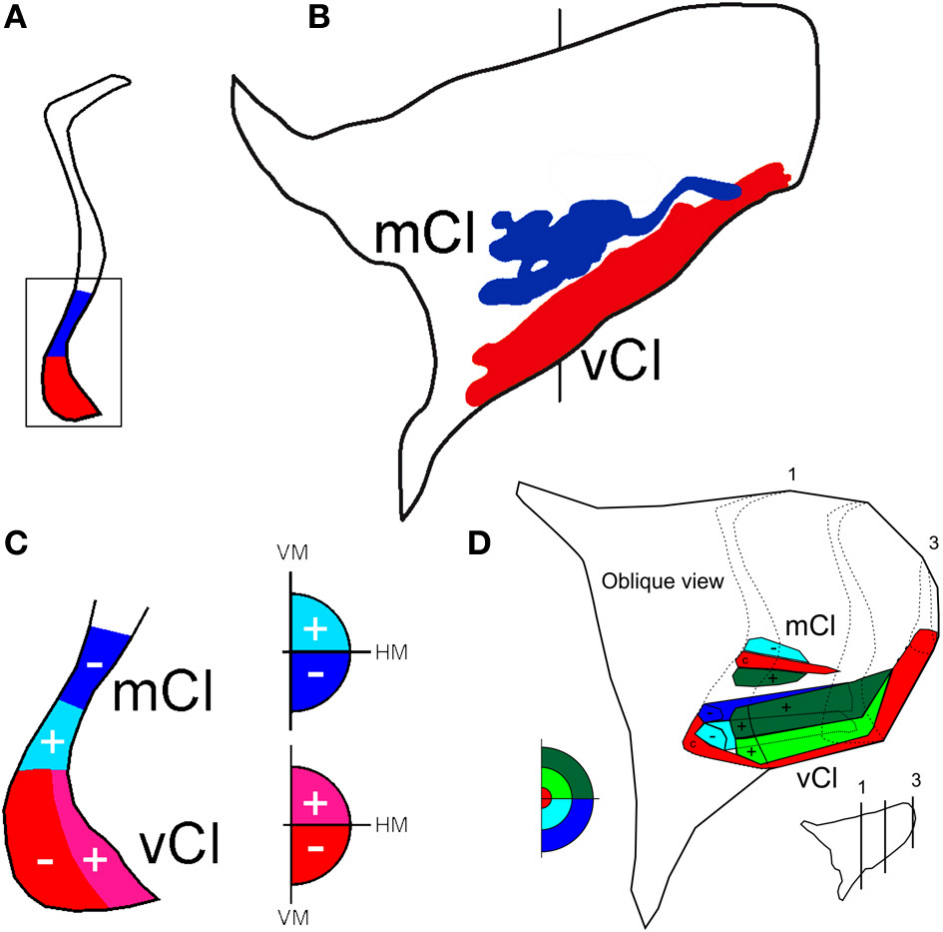
**Two topographically organized regions in the claustrum shown in a coronal section (A) at the level indicated in the lateral projection of the claustrum (B)**. The projections from the 19 injections in V4 were superimposed and are shown in the lateral reconstruction: mCl (blue) and vCl (red). The visual topography of these regions is shown in a representative coronal section in **(C)** and in an oblique reconstruction in **(D)**. c, indicates central field representation, (+) indicates the representation of the upper visual field, and (−) indicates the representation of the lower visual field in the claustrum.

The visual topography of the reciprocal connections of V4 with vCl and mCl in 19 cases is explained in Figure 7 and summarized in Figure 8. The connections found in these two regions of the claustrum were consistently observed in all animals studied, and both subdivisions appeared to have at least a coarse visuotopic organization in each area.

Figure 7 shows an intermediate level of summary data presentation between the 19 individual cases and the schematic topography shown in the next figure (Figure 8). This figure is built on the connections of V4 in 12 out of 19 injections in three sections at different levels of the claustrum. At the more anterior level, where the connections are more segregated and it is easier to observe a crude visuotopic organization with the upper field represented laterally in vCl and ventrally in mCl (Figure 7, section 1). At this level, the upper field injections corresponding to Cases 5 and 6, (Figures 6C, 3) show labeling in the lower portion of mCl and in the lateral portion of vCl (dark green, Case 6) and in the ventral lateral portion of vCl (light green, Case 5). Case 6 labels both areas (see Figure 3), but Case 5 only labels vCl (Figure 6C). Injections in central, intermediate and peripheral lower visual field representations in V4 label sequentially portions of vCl and mCl at this level. At a more posterior section (Figure 7B, section 2), projections are restricted to vCl and are more superimposed. Nonetheless, it is always possible to see a central to peripheral trend as well as a segregation of lower and upper field representations. As before, the upper field is represented laterally while the lower field is represented more medially. There is a larger emphasis of the central field representation that predominates in the posterior portion of vCl. Connections at more posterior levels are more superimposed and intermingled suggesting that the topography is more complex at that level.

After 19 injections in V4, both of these regions showed labeled cells and terminals that occupied the ventral and mid-portions of the nucleus (Figure 8A). The lateral reconstruction of the nucleus shows two regions that are elongated in the anterior-to-posterior dimension (Figure 8B). In the more dorsal labeled mCl, the connections with V4's lower visual field were found dorsal to the connections with V4's upper visual field (Figures 8C,D). In the ventral labeled vCl, the visuotopic organization was less clear but there was a tendency for the connections with V4's upper visual field to be located laterally to the connections with V4's lower visual field (Figures 8C,D).

## Discussion

Extrastriate area V4 plays a key role in relaying information from V2 to higher-order areas in the inferior temporal cortex (areas TEO and TE) that are critical for object recognition (Ungerleider et al., 2008). In the present study, we examined the relationship between V4 and the claustrum, and compared these projections with those from other neocortical areas.

The claustrum is a thin, irregular, sheet-like neuronal structure hidden beneath the inner surface of the neocortex. We found extensive reciprocal connections between V4 and the ventral portion of the claustrum (termed vCl) that extended through at least half of the rostrocaudal extent of the structure (Gattass et al., 2014). Additionally, in approximately 75% of the cases, we found reciprocal connections between V4 and a more restricted region in the claustrum, which was located farther dorsal, near the middle of the structure (termed mCl). Both vCl and mCl appear to have a crude topographic organization, based on the visuotopic location of our injection sites.

**Figure 9:**
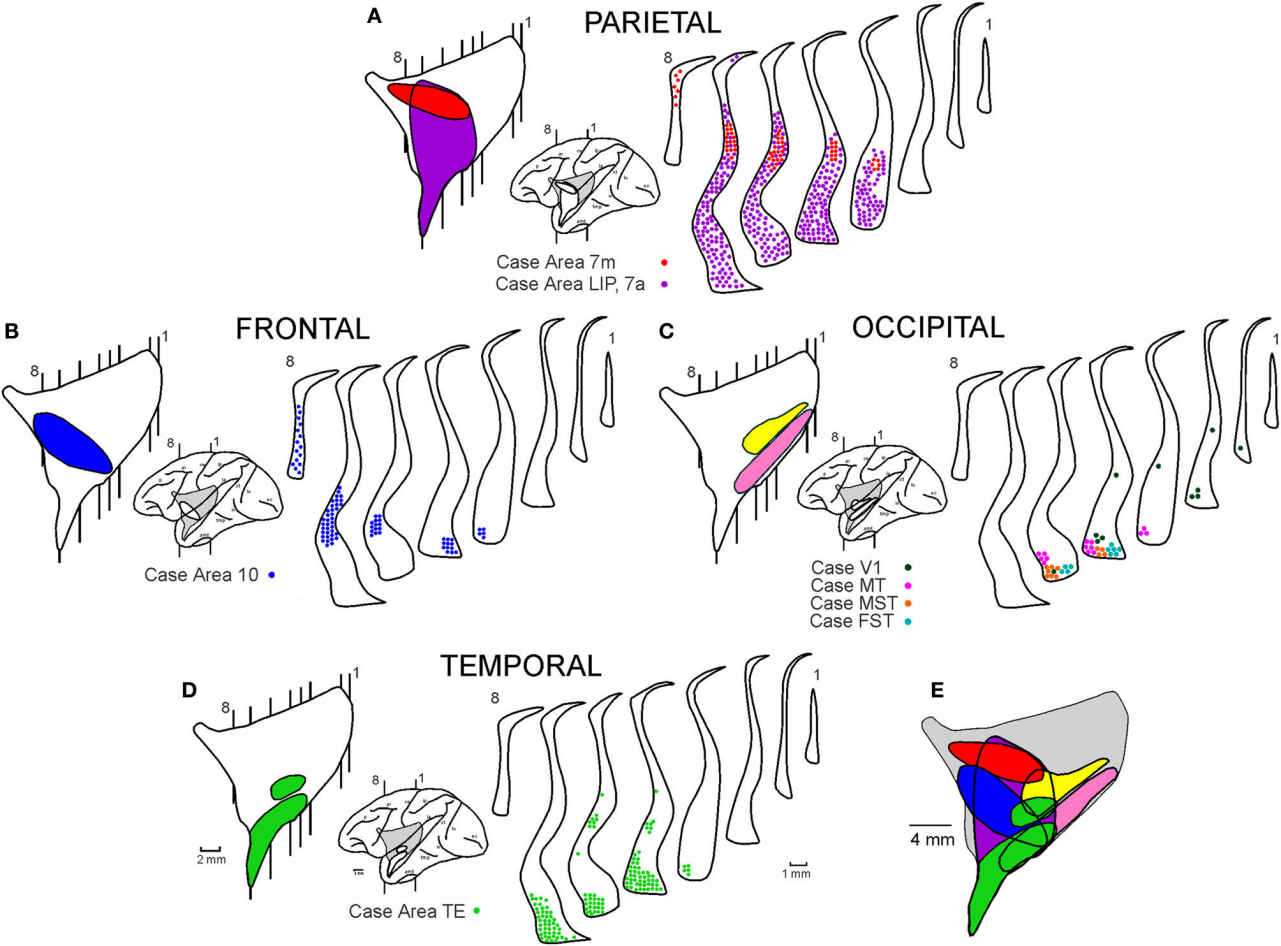
**Reciprocal connections of the claustrum with parietal (A), frontal (B), occipital (C), and temporal (D) areas**. All connections were superimposed in the lateral reconstruction of the claustrum **(E)**. Data were compiled from previous publications (Ungerleider et al., 1984; Boussaoud et al., 1992; Baizer et al., 1993; Webster et al., 1993) or presentations (Ribeiro Gomes et al., 2013). For details, see text.

Figure 9 shows existing data on the connections of the claustrum, which we summarize from previous publications (Ungerleider et al., 1984; Boussaoud et al., 1992; Baizer et al., 1993; Webster et al., 1993) or presentations (Ribeiro Gomes et al., 2013). The portions of the claustrum connected with V4 appear to overlap considerably with the portions connected with other visual cortical areas, including V1 (Mizuno et al., 1981; Doty, 1983), V2 (Pearson et al., 1982), MT (Maunsell and van Essen, 1983; Ungerleider et al., 1984), MST and FST (Boussaoud et al., 1992), TEO (Webster et al., 1993), and TE (Nauta and Whitlock, 1956; Kemp and Powell, 1970; Turner et al., 1980; Baizer et al., 1993; Webster et al., 1993). The regions of the claustrum connected with V4 and with occipital areas are illustrated in pink and yellow in the lateral reconstruction of the nucleus (Figure 9C). The connections with V1 (Ribeiro Gomes et al., 2013), MT (Ungerleider et al., 1984), MST, and FST (Boussaoud et al., 1992) are illustrated in the coronal sections of the claustrum in Figure 9C. Injections in TEO (Webster et al., 1993) and TE (Baizer et al., 1993; Webster et al., 1993) revealed connections with the anterior portion of mCl and VCl, which extends into the temporal branch of the claustrum (Figure 9D). Injections in the frontal pole of cortex (Brodmann area 10, Ribeiro Gomes et al., 2013) revealed connections with the anterior portion of vCl which extends to the frontal branch of the claustrum (Figure 9B). Injections in the parietal lobe (Baizer et al., 1993; Ribeiro Gomes et al., 2013) revealed extensive connections with the anterior and ventral portions of the claustrum (Figure 9A). The injection of Ribeiro Gomes et al. (2013) was placed on the medial surface in area 7 m and revealed a smaller connection than that of Baizer et al. (1993) who removed the medial bank of the intraparietal sulcus and placed injections into the lateral bank of the intraparietal sulcus that included LIPv and LIPd, extending into VIP and area 7a. These lateral intraparietal sulcus injections revealed extensive connections with the anterior and ventral portions of the claustrum. Figure 9E shows the plot of the connections revealing a crude topographic segregation, with a considerable amount of overlap, roughly obeying the “proximity” principle, whereby a given cortical region projects to the portion of the structure that is physically closest to it (Kemp and Powell, 1970).

The scheme proposed in Figure 9E is compatible with the data presented in this research topic by Reser et al. (2014) for the connections of the frontal pole with the claustrum in *Cebus apella*. The connections of areas 9, 10, and 12 of the frontal lobe are clustered in areas vCl and mCl, in addition to an area located dorsally and anteriorly that they termed fCl (Figure 6B of Reser et al., 2014). This area (fCl) is comparable to the frontal subdivision shown in Figure 9B (Ribeiro Gomes et al., 2013).

Evidence in other species suggests that the claustrum may be specialized for visuomotor function due to of its connections with different visual and motor subdivisions of cortex (Olson and Graybiel, 1980). Based primarily on findings from a study using 2-DG, Ettlinger and Wilson (1990) speculated that the claustrum is involved in cross-modal associations.

The present results are consistent with a mixed organizational scheme, namely, a ventral visuotopic projection system combined with a dorso-ventral and anterior-posterior connectional system segregated with some degree of topographical proximity. Thus, in addition to the visuotopic subdivisions we describe here, the anatomical connections of the claustrum resemble those of the caudate nucleus, which obeys the “proximity” principle (Kemp and Powell, 1970). According to this principle, the frontal cortex would project to the head of the caudate, the parietal cortex to the body, the occipital cortex to the genu, and the temporal cortex to the tail. This organizational scheme differs somewhat from that proposed by Saint-Cyr et al. (1990) for the caudate, who found that the projection strips arising from cortical visual areas are limited in length, and thus show some degree of topographic proximity.

**Figure 10:**
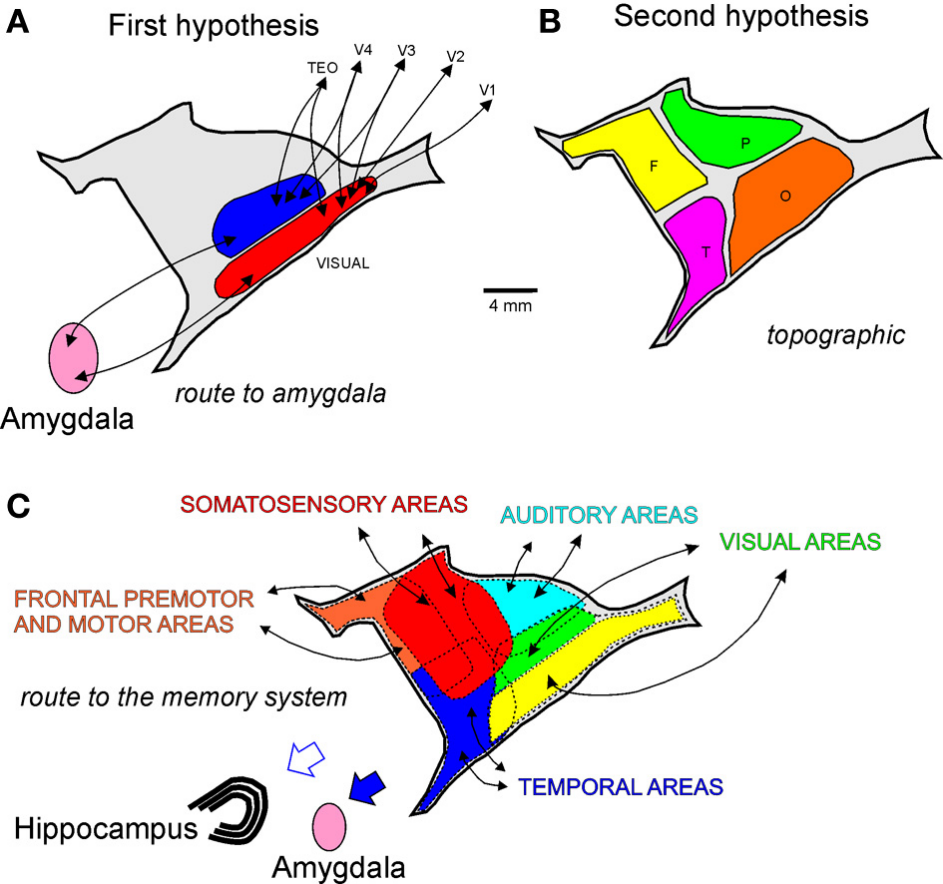
**Hypothetized anatomical organization of the claustrum**. Lateral reconstructions of the claustrum with pictorial representations of three connectional hypotheses: **(A)** *route to the amygdala*; **(B)** *topographic* (O, occipital; P, parietal; T, temporal; and F, frontal connections); and **(C)** *route to the memory system*.

Based on our data and other connectional studies of the claustrum (Ungerleider et al., 1984; Boussaoud et al., 1992; Baizer et al., 1993; Webster et al., 1993; Ribeiro Gomes et al., 2013), we propose three hypotheses for the connectional organization of this nucleus. Figure 10 is a pictorial representation of these three hypotheses. The first hypothesis addresses the two visuotopically organized areas reciprocally connected with visual cortical areas and projecting to the amygdala. This hypothesis is based on the connectional data of V4 and other visual areas. We termed this hypothesis “route to amygdala,” which potentially carries information about objects in visual space (Figure 10A). The second hypothetical scheme is topographical, whereby reciprocal connections of the different neocortical lobules are topographically segregated in the claustrum (Figure 10B). In spite of the large degree of overlap, double-labeled cells projecting to the temporal and parietal cortices from the claustrum are virtually nonexistent (Baizer et al., 1993). The third hypothetical scheme (Figure 10C) incorporates the findings of Milardi et al. (2013) described in the next paragraph. It is clear that the first and the third hypothetical scheme are compatible and that they coexist in the claustrum. The work of Reser et al. (2014) for the connections of the frontal pole with the claustrum in *Cebus apella* in this *research topic* shows that injections in visual-related areas of the frontal cortex label the fCl region (*by topographical proximity*) but also label the visual related areas vCl and mCl (*as a route to the amygdala*). It is worth noticing that neurophysiological recording in the claustrum revealed mostly unisensory responses (Remedios et al., 2010), a finding consistent with the absence of double labeling found in the claustrum (Baizer et al., 1993). The homogeneous cell type of the claustrum may convey unisensory information, but the interaction between neighboring cells may be important for multimodal integration in this structure.

Using constrained spherical deconvolution tractography, Milardi et al. (2013) described four groups of white matter fibers that connect the claustrum to the cortex. The anterior and posterior cortico-claustral tracts connect the claustrum to the prefrontal cortex and visual areas. The superior tract link the claustrum to the sensory-motor areas, while the lateral pathway connects the claustrum to the auditory cortex. Additionally, this study demonstrated a claustrum-medial pathway that connects the claustrum to the basal ganglia, specifically the caudate nucleus, putamen, and globus pallidus. Together, the first and the third hypothetical schemes best represent our current understanding of claustral connectivity. We also consider the crude visual topography of mCl and vCl useful in organizing the visual connections of several visual cortical areas to the claustrum.

### Conflict of interest statement

The Review Editor David A. Leopold declares that, despite being affiliated to the same institution as authors Robert Desimone and Leslie G. Ungerleider, the review process was handled objectively and no conflict of interest exists.
